# Cascaded 3D UNet architecture for segmenting the COVID-19 infection from lung CT volume

**DOI:** 10.1038/s41598-022-06931-z

**Published:** 2022-02-23

**Authors:** Aswathy A. L., Vinod Chandra S. S.

**Affiliations:** Department of Computer Science, University of Kerala, Thiruvananthapuram, India

**Keywords:** Image processing, Diseases

## Abstract

World Health Organization (WHO) declared COVID-19 (COronaVIrus Disease 2019) as pandemic on March 11, 2020. Ever since then, the virus is undergoing different mutations, with a high rate of dissemination. The diagnosis and prognosis of COVID-19 are critical in bringing the situation under control. COVID-19 virus replicates in the lungs after entering the upper respiratory system, causing pneumonia and mortality. Deep learning has a significant role in detecting infections from the Computed Tomography (CT). With the help of basic image processing techniques and deep learning, we have developed a two stage cascaded 3D UNet to segment the contaminated area from the lungs. The first 3D UNet extracts the lung parenchyma from the CT volume input after preprocessing and augmentation. Since the CT volume is small, we apply appropriate post-processing to the lung parenchyma and input these volumes into the second 3D UNet. The second 3D UNet extracts the infected 3D volumes. With this method, clinicians can input the complete CT volume of the patient and analyze the contaminated area without having to label the lung parenchyma for each new patient. For lung parenchyma segmentation, the proposed method obtained a sensitivity of 93.47%, specificity of 98.64%, an accuracy of 98.07%, and a dice score of 92.46%. We have achieved a sensitivity of 83.33%, a specificity of 99.84%, an accuracy of 99.20%, and a dice score of 82% for lung infection segmentation.

## Introduction

SARS-CoV-2 is the seventh member of coronavirus family, which has caused infections in humans^1^. Coronaviruses (CoV) are a large virus family that spreads fast from person to person. COVID-19 was declared as a Public Health Emergency of International Concern by the World Health Organization (WHO) on January 31, 2020^2^. According to the WHO’s weekly epidemiological update, there were 150,110,310 confirmed cases of COVID-19 up to April 30, 2021, with 3,158,792 deaths. Coronavirus is a hazardous virus spread through droplets in the air, intimate contact, aerosols, and touching infected surfaces. Now a day, there exist different variants of coronaviruses. In some cases, COVID-19 causes severe pneumonia in both lungs. The inflammation caused by the lung infection makes breathing difficult for the person. Consequently, individuals with severe infections require a ventilator. Since COVID-19 is a pandemic and there are limited hospital facilities, such as oxygen availability, ventilators, and doctors, it is imperative to diagnose and prognosis patients efficiently. To decide the prognosis, lung infections ought to be detected. By automating, we can decrease the death rate by increasing the speed of patient isolation, reducing the diagnosis time of patients, and arranging the necessary amenities such as oxygen and ventilator ahead of time.

Clinicians used a variety of methods to confirm COVID-19. The procedures include real-time Reverse Transcription Polymerase Chain Reaction (RT-PCR), nonPCR testing such as isothermal nucleic acid amplification technology^3^, non-contrast chest Computed Tomography (CT), and radiography^4^. RT-PCR is widely used to confirm COVID-19, but its sensitivity is limited^3^. Many diseases can now be diagnosed using digital imaging. Most doctors employ X-ray and CT imaging techniques to diagnose and prognosis COVID-19. In this study, CT images were used to monitor lung changes and detect infections. COVID-19 can be detected in X-ray images in a variety of ways. Aditya Borakati et al. conducted a study to examine the diagnostic accuracy of chest X-rays and CT scans in COVID-19-infected patients^5^. According to the study, CT has higher diagnostic accuracy than chest X-rays. As a result, CT should be considered in the initial evaluation of COVID-19.

The SARS-CoV-2 infection causes damage to the alveoli, our lungs’ tiny air sacs, and the surrounding tissues. This inflammation causes fluid deposit and dead cells in the lungs. This will impair oxygen transmission, resulting in symptoms such as shortness of breath and coughing. It may lead to death also. As a result, a medical support system is required for treatment. The precise segmentation of the diseased region is critical for determining the severity of the infection and planning the medical supportive (mechanical ventilation) system.

**Figure 1:**
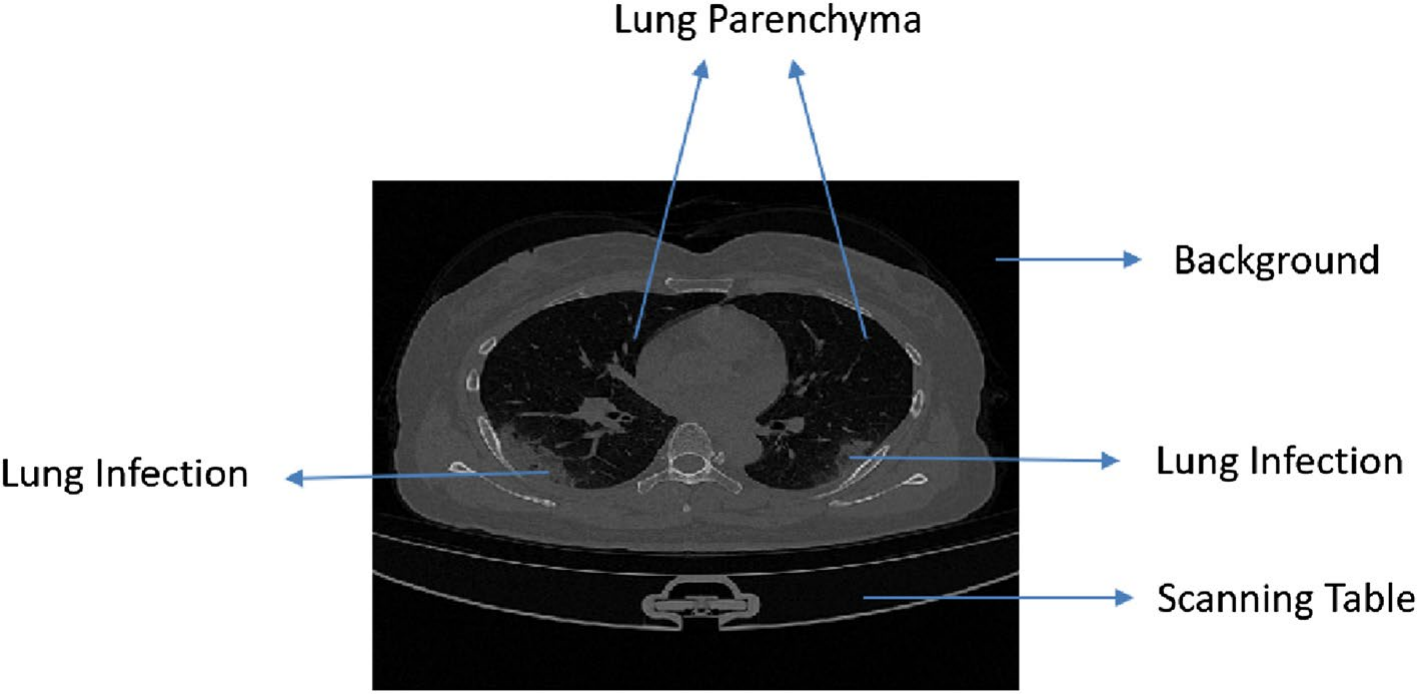
CT Image of Lung.

Deep neural network has a significant impact on medical image segmentation, especially in lung diseases. Figure 1 shows a CT scan of lungs infected with COVID-19. It shows lung parenchyma and lung infections. In this work, two-stage architecture is designed to segment the infected region from the lungs: the first stage deals with the lung region, and the second stage deals with the lung infection. To detect malignant lung nodules, El Bana et al.^6^ created an automated two-stage framework with the concept of semantic segmentation by using deepLab-V3 plus model. Two Convolutional Neural Network (CNN) based models for identifying lung cancer on lung CT images were proposed by Polat et al.^7^. A straight 3D CNN with softmax and a hybrid 3D CNN with the Radial Basis Function based Support Vector Machine (SVM) is used as the classifier. The proposed hybrid 3D CNN network with SVM provides better performance of lung cancer diagnosis, with 91.81% accuracy, 88.53% sensitivity, and 91.91% precision. Nasrullah et al.^8^ proposed a multiple strategy-based automated lung nodule detection. Two 3D convolutional networks are used to detect lung nodules and classify them. A Faster R-CNN with CMixNet is used for nodule identification, and the Gradient Boosting Machine (GBM) with 3D CMixNet is employed for classification. On the LIDC-IDRI data set, they got a sensitivity of 94% and a specificity of 91%.

Once the lung parenchyma for the corresponding input volume is detected, then we have to segment the lung infection from these lung regions. Lung infection region segmentation is an important task since the lung contains pulmonary vessels, aorta, etc. The related works are explained in “Related works”. Most of the works used complicated CNN architecture to perform the lung infection segmentation. Since we are going through a pandemic situation, the accurate segmentation of lung infection helps doctors to suggest the correct life-saving methods for high-risk patients. To achieve this, we incorporated the basic image processing techniques with the deep learning architecture.

With the usage of the deep architecture, there is no need to label the lung parenchyma for new COVID-19 CT volumes. With the available small data set, the basic image processing techniques, and the advanced CNN models, we improved the accuracy. Using the simple architecture for deep learning, we were able to reduce the number of parameters and complications. We extracted the ROI with the help of proper pre-processing; hence, the 3D UNet can learn more efficiently by narrowing down the lung’s parenchyma volume. The accuracy improved as a result of the step-by-step learning process. The main contributions are

1. We developed a step-by-step framework to segment the lung parenchyma from the input 3D CT volume and the infected lung region due to COVID-19 from the lung parenchyma.
2. An appropriate pre-processing is carried out so that the regions which are similar to the background can be removed before giving to the 3D UNet for training. The 3D UNet can learn more efficiently by narrowing down the lung’s parenchyma volume.
3. By Utilizing the basic image processing principles and the simple deep learning architecture, reduced the number of parameters of the deep architecture and obtained a better accuracy.
4. The doctors can see the infected region based on the intensity of the infection with the help of a colormap in the entire 3D volume. So it can be treated as a supporting tool for the doctors.

## Related works

Many works were reported for the lung infection segmentation due to COVID-19 by using the deep learning architectures.

Zheng et al.^9^ developed a weak supervised software to identify COVID-19. A pre-trained UNet generates the lung mask, and it is input into the deep learning architecture DeCoVNet. The DeCoVNet consists of three stages—network stem, residual block, and progressive classifier to predict the likelihood of COVID-19 infection. They acquire a specificity and sensitivity of 0.911 and 0.907 using a probability of 0.5 to classify whether it is COVID positive or negative. Zhou et al.^10^ proposed a UNet segmentation network based on an attention mechanism. The feature representations from the encoder are given as input to the attention mechanism. The channel-wise and space-wise reweighting of these features are performed in the attention mechanism, thereby getting the most prominent features. Then these features are projected to the decoder part. To address the small lesion segmentation, focal tversky loss function is used. Jin et al.^11^ suggested a classification and segmentation system for COVID-19. They used the 3D UNet++ and ResNet-50 combined model to achieve a better result. Amayar et al.^12^ explored the encoder and decoder architecture for detecting and segmenting the infected lesions from the chest CT. This work follows a multi-task learning approach to COVID-19 classification, segmentation, and reconstruction.

The Joint classification and segmentation system proposed by Wu et al.^13^ consists of an explainable classification system to detect COVID-19 opacifications and another pipeline to segment the opacification areas. Fan et al.^14^. proposed an architecture called Inf-Net, which consists of an edge attention module, parallel partial decoder, and reverse attention module. Then proposed a Semi-Supervised Inf-Net, to address the limited number of training samples. Yan et al.^15^ designed a deep CNN, COVID-SegNet for COVID-19 infected lung region segmentation. COVID-SegNet has two parts: an encoder and decoder. Feature variation and a progressive atrous spatial pyramid pooling are added to get important features. The residual blocks are used to avoid the gradient vanishing problem. Wang et al.^16^ proposed a COVID-19 Pneumonia Lesion segmentation network (COPLE-Net) to learn from the noisy images. Aswathy et al.^17^ developed a transfer learning method to diagnose COVID-19 and determine its severity from CT images. Nature-inspired optimization techniques have been explored in COVID-19 classification, and severity prediction by Suma et al.^18^. Feature extraction with different pre-trained networks and various classifiers are also investigated by Aswathy et al.^19^. Pang et al.^20^ devised a mathematical model to find the intervention and monitoring of COVID-19, infectious disease. The work represents a collaborative city, Digital Twin, with federated learning that can learn a shared model by retaining the training data. It can lead to a better solution by acquiring knowledge from multiple data sources. The analysis and the prediction of COVID-19 spread in India are studied by Kumari et al.^21^. Singh et al.^22^ proposed a method to diagnose COVID-19 from chest X-ray images. The method incorporates the wavelet decomposition to get multiresolution of the input image, and it considers three classes normal, viral pneumonia and COVID-19. Cascaded Generative Adversarial networks are also used for detection problems in many computer vision algorithms^23^.

## Methodology

### Data set

We have used the public data set given by Ma et al.^24^. The dataset comprises 20 COVID-19 CT 3D volumes that have been labeled. The left lung, right lung, and infected regions are labeled by two radiologists and verified by an experienced radiologist. The Coronacases Initiative and Radiopaedia provided the CT scans in NIfTY format, which were licenced under CC BY-NC-SA. The lowest CT volume in this dataset is 630 × 630 × 36, while the largest CT volume is $630\times 630\times 418$.

**Figure 2:**
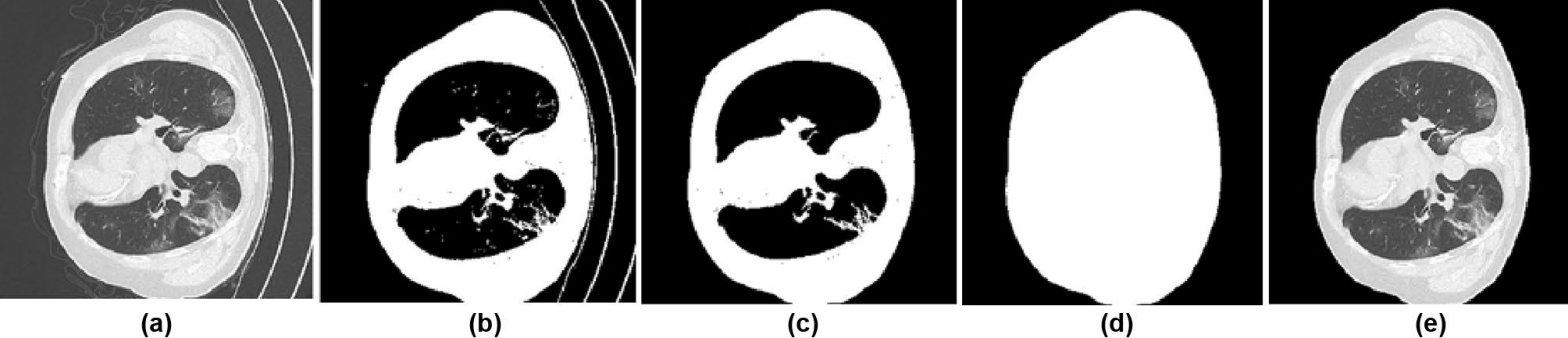
Different steps in Preprocessing.

### Preprocessing

The first ten volumes in this data collection have a resolution of 512 $\times$ 512 and a varying number of slices. The last ten volumes are from the Radiopaedia, each with a different number of slices and a resolution of 630 $\times$ 630. While taking the CT, the input images contain some unwanted information. As a result, preprocessing is essential to eliminate such unwanted information from the volumes. The size of each slice in the 3D CT data is decreased to 256 $\times$ 256 to reduce the memory usage. According to the study by Olisah et al.^25^, preprocessing can increase CNN accuracy. The preprocessing consists of the following steps 
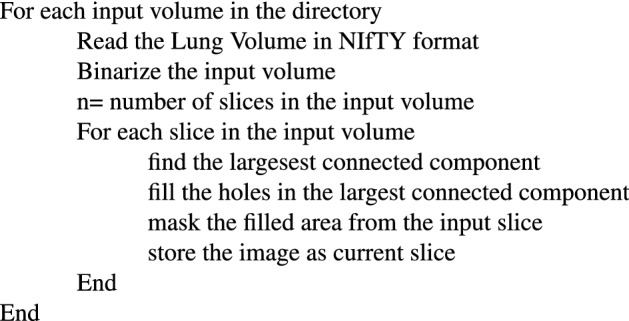


By this, we can make the background to zero and eliminate the same intensity present outside the lung parenchyma. Figure 2 shows the different steps involved in preprocessing the input volumes. Figure 2a shows a sample input slice, Fig. 2b shows the binarized image, Fig. 2c shows the largest connected component and Fig. 2d shows the filled image and Fig. 2e shows the corresponding portion from the input image. Thus, eliminating unwanted portions from the input volume.

### Patchwise augmentation

Because the inputs are 3D volumes, a large amount of GPU memory is required. We employed a 3D patch-based technique to reduce this. Each input volume is divided into 16 patches of size $128\times 128\times 32$ randomly. A well-trained CNN must be invariant to translation, size, and viewpoint. To achieve this, a large amount of input data is required. We used augmentation to achieve an extensive amount of data and obtained the data invariance. The augmentation was performed in a random order to each of the $128\times 128\times 32$ patches. Rotating at $90^{\circ }$, horizontal and vertical translations, zooming, and shearing are some of the augmentation techniques used in this work.

### Overview of the proposed method

**Figure 3:**
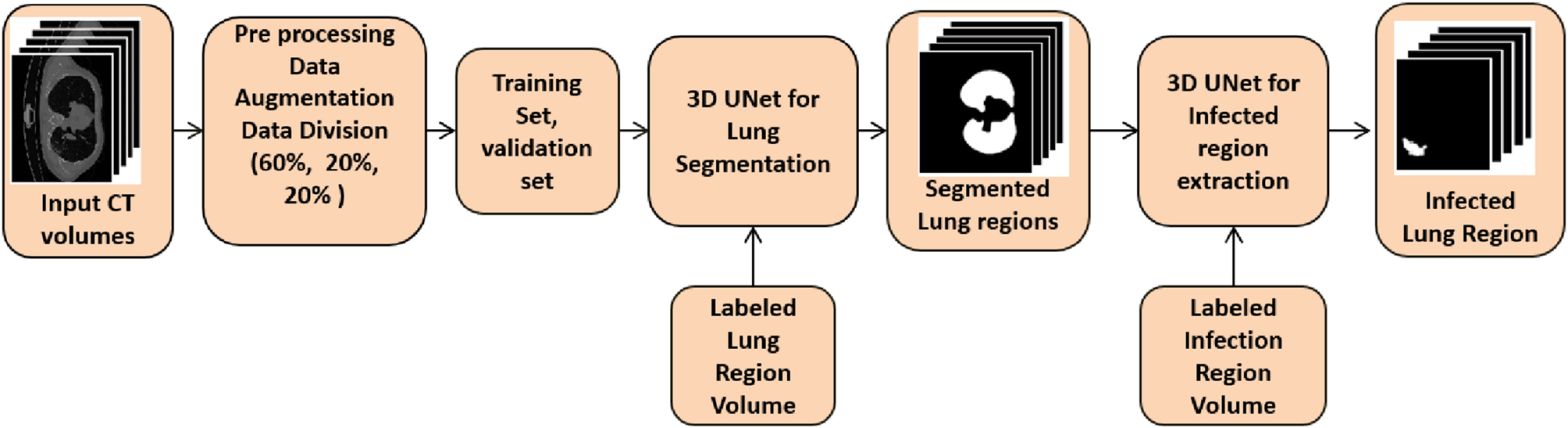
Overview of the proposed system architecture.

We employed a simple 3D UNet design to learn the lung parenchyma and the infected lung area independently, rather than employing complicated deep learning architectures. The overall framework of the proposed technique is shown in Fig. 3. The input CT volumes are first pre-processed, then separated into three groups: 60% for training, 20% for validation, and 20% for testing randomly. The patches are extracted, the augmentation is applied, and the data is fed into the initial 3D UNet architecture^26^ to learn the lung parenchyma volume for training and validation. Only the cropped parenchyma volumes are sent to the second 3D UNet after post-processing to reduce the number of background pixels. The second 3D UNet segments the infected lung area from these lung parenchymas.

#### Segmentation of the lung region

**Figure 4:**
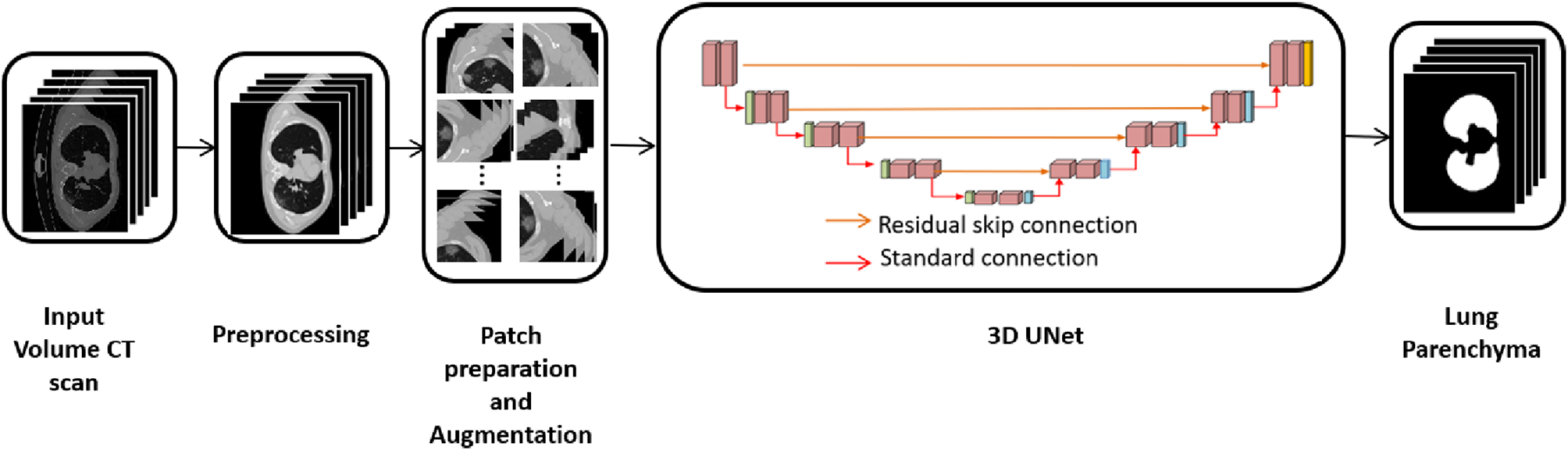
Lung parenchyma segmentation architecture.

Since lungs are part of the respiratory system and their primary function is to transport oxygen into the bloodstream, for any lung diseases such as lung cancer, cystic fibrosis, Acute Respiratory Distress Syndrome, early detection is an essential factor. It is challenging because the lung appears different from the normal view if a patient has any lung diseases. The texture, shape, and colour of the lung parenchyma may change as a result of infection or disease. Several lung segmentation methods exist, including thresholding, region-based methods, graph cuts, shape-based methods, and so on. The segmentation of the lung parenchyma is also aided by machine learning. In this work, lung region is segmented using the 3D UNet method. The detailed architecture of the Lung parenchyma segmentation is shown in Fig. 4.

**Figure 5:**
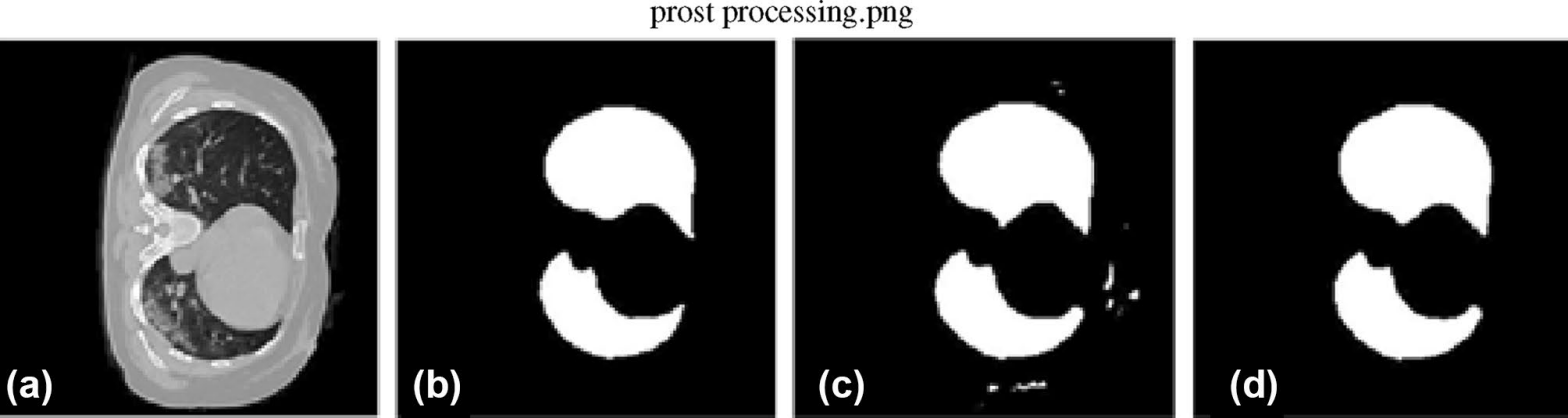
Post processig after lung parenchyma segmentation.

The augmentation is applied and input CT volumes inputted to the first 3D UNet after the patch extraction from the preprocessed data. It consists of an analysis path and a synthesis path. It includes the convolution layer, pooling layer, activation function, up-convolution layer, and concatenation layer, as shown in Fig. 4. The convolutional layer is the basic block of CNN, and it extracts image features by using a number of kernels. A nonlinear activation function maps these extracted features. The output of each neuron in the CNN is determined using a Rectified Linear Unit activation function, which is a nonlinear activation function (ReLU). When compared to the Sigmoid and tanH functions, it has better performance, faster learning, and a simpler structure.

Pooling is used to downsample the feature maps and merge related features in the convolutional layer. The pooling operation can also reduce the number of parameters processed by the CNN. Normally, there is maxpooling, which gives the most valued feature, and average pooling, which gives the feature’s average presence. In the 3D-UNet, the analysis phase, each layer is equipped with two $3\times 3\times 3$ convolutional layers, each followed by a ReLU activation and a $2\times 2\times 2$ maxpooling with a stride of two is used.

The synthesis path consists of transposed convolutions and a concatenation layer. The transposed convolutional layer, also known as the deconvolutional layer, produces a spatial dimension of the same size. In the analysis phase, concatenating the up convolved feature maps from the lower layer and feature maps of the same resolution will produce more features. The concatenation layer will do this. A $1\times 1\times 1$ convolution in the final layer lowers the number of output channels to the number of labels, which in our case is two. The architecture has a total of 19,069,955 parameters.

The input to the network is a volume of $128\times 128\times 32$ voxels, and output in the final layer is $128\times 128\times 32$ voxels in x, y, and z directions, respectively. We used a batch size of eight and a learning factor of 0.0005 for lung parenchyma segmentation. The number of epochs used is 20. The validation frequency is set to 50, and the optimizer used is Adam. The CNN got a validation accuracy of 97.35%.

Because we only have a limited number of CT volumes and distinct intensity features, the 3D CNN will detect certain small other regions in some CT slices instead of the lung parenchyma. We can filter out these unwanted portions by taking the two largest connected components. The process is shown in Fig. 5. Figure 5a shows a slice of the input CT volume, Fig. 5b shows the ground truth, and Fig. 5c shows the corresponding slice output from the first 3D UNet. Since some regions are more similar to the lung parenchyma or the infections present in the lung parenchyma, these regions are also captured by the 3D UNet. Figure 5d shows the two largest connected component from the output slice.

**Figure 6:**
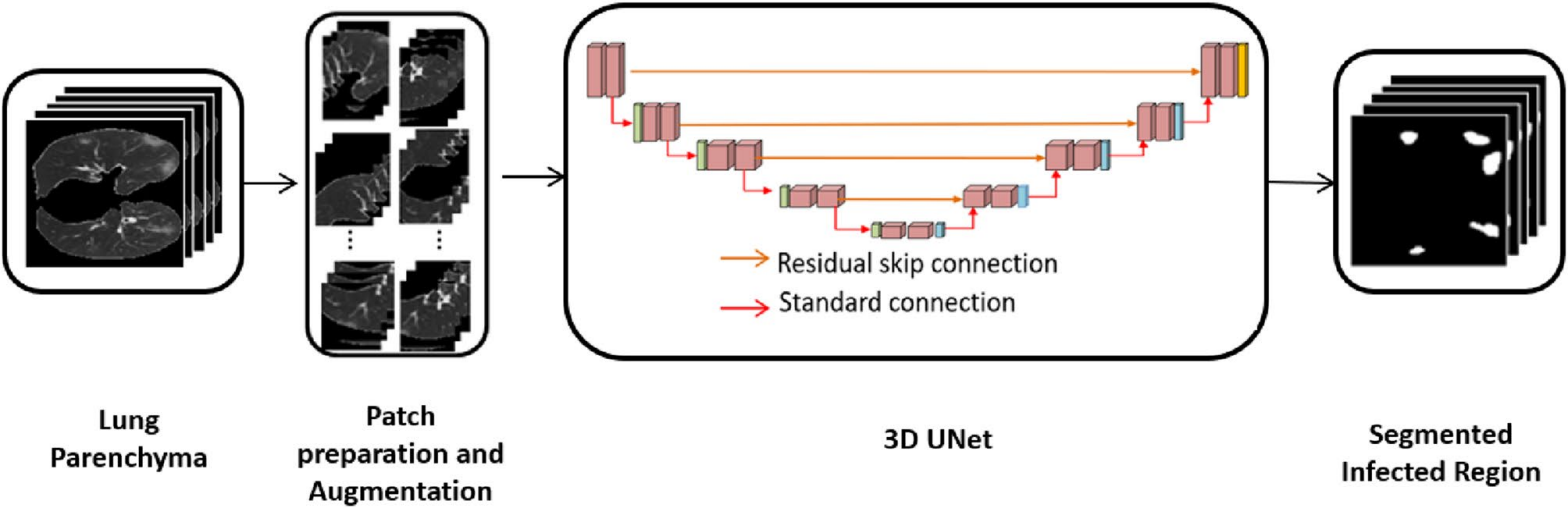
The proposed architecture for lung infected region.

#### Segmentation of the lung infected region

Many studies on the infected lung area extraction have been reported. Before inputting the segmented parenchyma to the second 3D UNet, we find the largest bounding box that can fit within the current CT volume, which we acquired from the first 3D UNet. This will limit the number of background pixels to be processed or learned. Therefore, we can reduce the number of background pixels by maintaining only that much 3D volume. Figure 6 shows the workflow for segmenting the infected lung region in the second 3D UNet. After the cropping of the lung parenchyma volume, patches are created, augmentation is applied, and these patches are given as input to the second 3D UNet. Here also the patch size used as $128\times 128\times 32$ voxels in x, y, and z directions. For each input volume, 32 patches are extracted randomly, and augmentation is applied to each patch. The output patch size is $128\times 128\times 32$ voxels in x, y, and z directions, respectively. The output of the 3D UNet will be the infected region. We used a batch size of eight, a learning factor of 0.0005, Adam as the optimizer and a total of 20 epochs.

**Figure 7:**
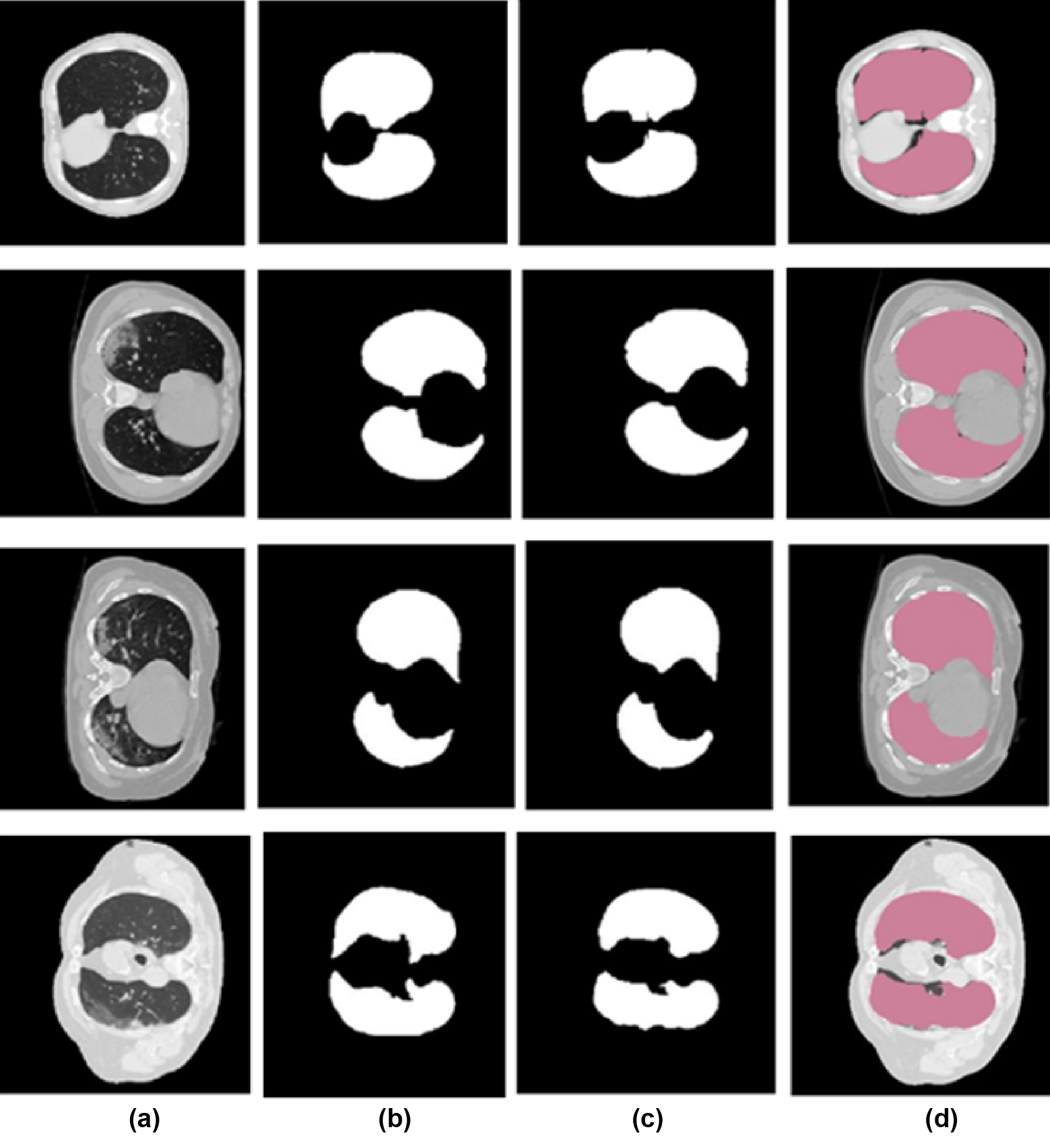
The output of lung parenchyma segmentation. (**a**) Input image slice, (**b**) ground truth, (**c**) proposed method output, (**d**) overlayed image.

### Ethics approval

This article does not contain any studies with human participants or animals performed by any of the authors.

## Results and discussion

We compare the proposed method with various methods in the current literature in this section. The first section will discuss the results of lung parenchyma segmentation, which can be utilized as a framework for detecting any lung abnormalities. The second section compares the proposed infection region segmentation to the approaches in the current literature.

### Performance metric

To do the segmentation analysis with the current methods, we choose the following performance metric based on the True Positive (TP), True Negative (TN), False Positive (FP), and the False Negative (FN).

1 $$\begin{aligned}&Specificity=TN/(TN+FP). \end{aligned}$$

2 $$\begin{aligned}&Sensitivity =TP/(TP+FN) . \end{aligned}$$

3 $$\begin{aligned}&Accuracy= (TP+TN)/(TP+TN+FP+FN) . \end{aligned}$$

4 $$\begin{aligned}&Precision =(TP)/(TP+FP). \end{aligned}$$

5 $$\begin{aligned}&Dice= (2*TP)/(2*TP+FP+FN). \end{aligned}$$

6 $$\begin{aligned}&MCC=(TP*TN-FP*FN)/sqrt((TP+FN)*(TP+FP)*(TN+FP)*(TN+FN)). \end{aligned}$$

### Lung parenchyma segmentation method

For Lung parenchyma segmentation, the proposed method got a validation accuracy of 97.35%. Figure 7 shows the results of some slices in the dataset. Figure 7a shows a slice of the input CT volume, Fig. 7b shows the ground truth, Fig. 7c shows the results we obtained for the corresponding input volume using the proposed method, and Fig. 7d shows the the relayed image of the input image and the output of the proposed work. By evaluating the experiments, we found that the proposed parenchyma segmentation preserves both accuracy and precision.

Even though many lung parenchyma segmentation methods use both 2D and 3D data, a precise comparison is not possible. We conducted different studies based on the different epochs and different patch sizes. By analyzing, it is observed that the input and output patch size with $128\times 128\times 32$ gave the best results for the lung parenchyma segmentation.

**Figure 8:**
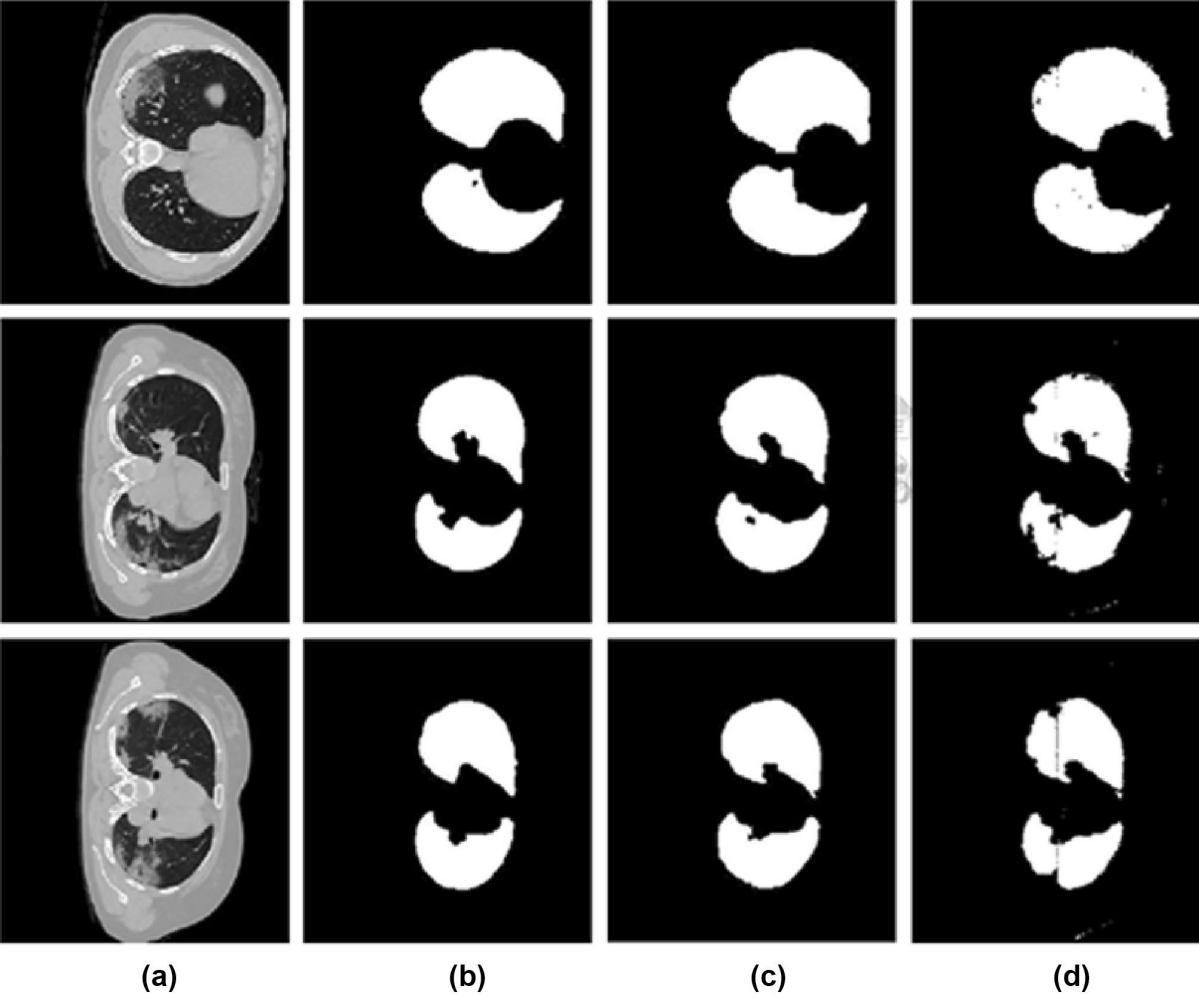
Comparison of Adam and Sgdm optimizer in lung parenchyma segmentation. (**a**) Input image slice, (**b**) ground truth, (**c**) output with Adam optimizer, (**d**) output with Sgdm optimizer.

We used both the Adam optimizer and the Sgdm optimizer to identify the best optimizer for this work. The adding up of momentum in the stochastic gradient descent helps to accelerate in the proper direction. Adam is an adaptive learning rate method and is a combination of RMSprop and Stochastic Gradient with Momentum. It adjusts the learning rate for each network weight based on the first and second moments of the gradient.

Figure 8 illustrates some of the output CT slices obtained using the Adam optimizer and the Sgdm optimizer from the proposed architecture. Slices of the input 3D volume are shown in Fig. 8a. The ground truth is shown in Fig. 8b. The output from the proposed method is shown in the Fig. 8c. Figure 8d displays the corresponding slices obtained by utilising the Sgdm optimizer with proposed method. The Sgdm fails to detect the lung pixels, especially at the boundary, as can be seen in the output images. According to the comparison results, the Adam optimizer performs the best, and we can also retrieve all of the pixels in the boundary by employing it.

We analyzed the proposed lung parenchyma segmentation performance with different network settings like the number of epochs, different optimizers, and different initial learning rates. Table 1. shows the number of epochs, corresponding validation accuracy, and the testing accuracy with initial learning rate 0.0005 and the Sgdm optimizer. By analyzing, it is observed that at epoch 20 with learning parameter 0.0005, the network got a validation accuracy of 97.35% and an accuracy of 98.07% with the SARS-CoV-2 CT data set, which is a comparably promising result.

**Table 1:** Accuracy for lung parenchyma segmentation with different epochs.

No. of epochs	Validation accuracy (%)	Testing accuracy (%)
10	94.26	93.13
15	95.63	95.28
20	97.35	98.07

### Infected region segmentation

We conducted experiments using various patch volume sizes, optimizers, and iterations for the infected lung region segmentation. Table 2 illustrates the number of epochs, related validation accuracy, and testing accuracy for lung infected region segmentation with an initial learning rate of 0.0005 and Adam optimizer.

**Table 2:** Accuracy for lung infection segmentation with different epochs.

No.of epochs	Validation accuracy (%)	Testing accuracy (%)
10	89.47	90.65
15	92.71	94.28
20	96.83	99.20

**Figure 9:**
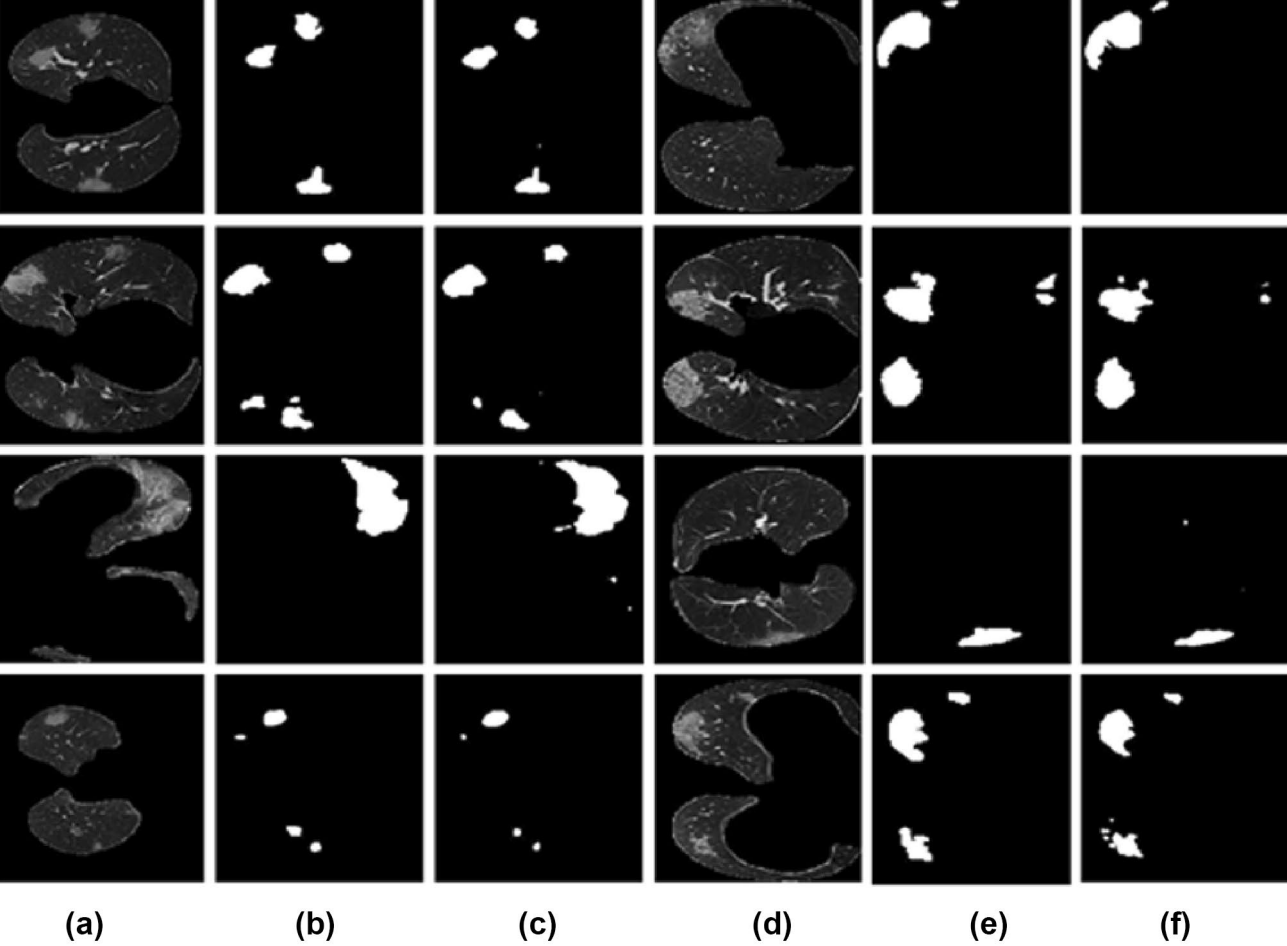
Visual comparison of COVID-19 infection segmentation results against GT. (**a**,**d**) The input image slices, (**b**,**e**) the ground truth, and (**c**,**f**) the output obtained from the proposed method.

The visual comparison of some of the input slices to the ground truth is shown in Fig. 9. Column (a) and (d) shows the input slice, column (b) and (e) shows the ground truth, column (c) and (d) shows the output we got from the proposed method. By seeing the results, it is clear that the proposed method is able to detect even small infections very accurately.

**Figure 10:**
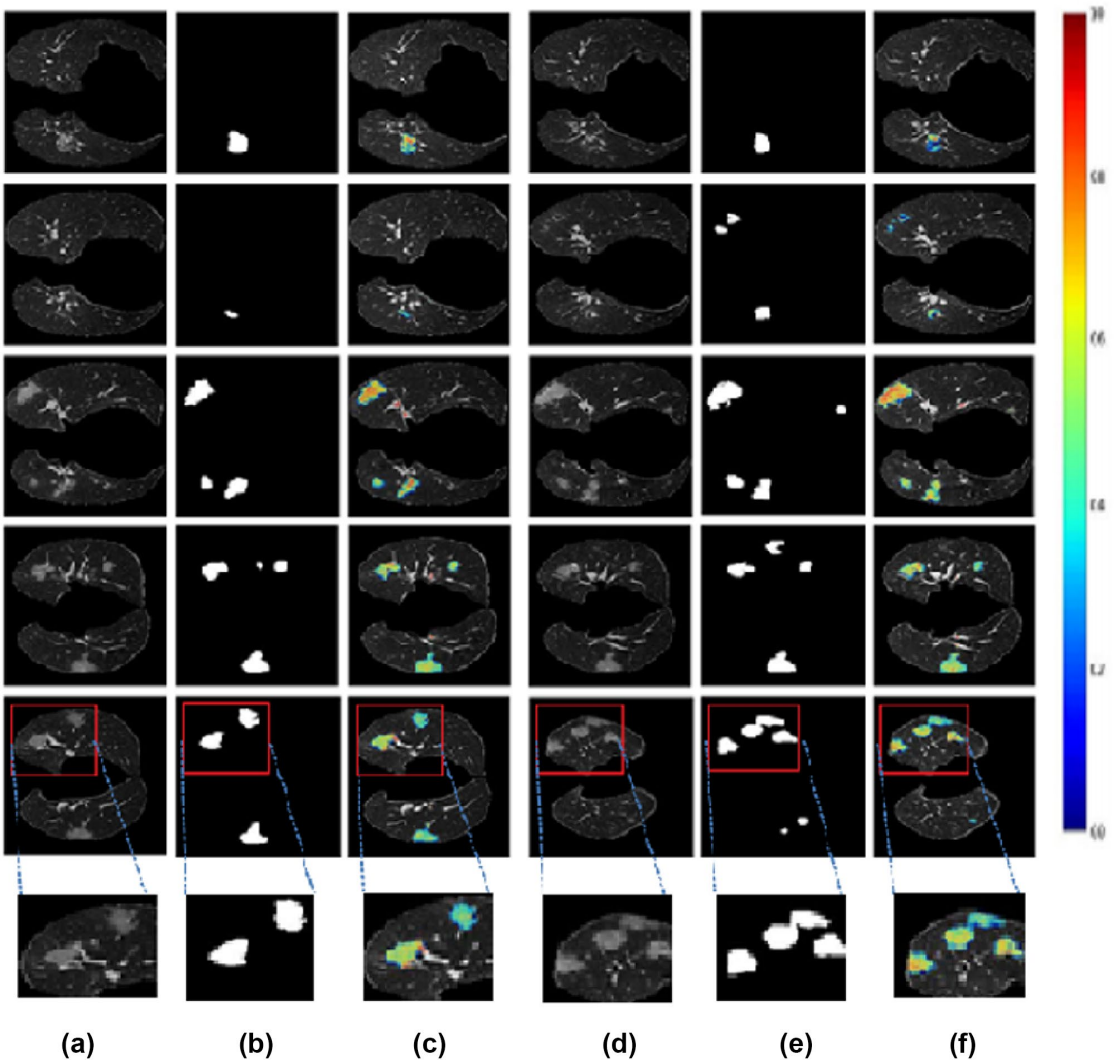
Output results with colormap.

By examining each of the slices, it is evident that the proposed network captures most infections. Even though the arteries have the same intensity values, the proposed method is able to detect lung infections correctly. According to the results, the infection region network trained at the 20*th* epoch with $128 \times 128\times 32$ patches and Adam optimizer with learning rate 0.0005 performed better. Here we got a validation accuracy of 96.83%. The colormap-based infection representation of the CT slices obtained from the second 3D UNet is shown in Fig. 10. The colormap ‘jet’ is used here. The range of pixel values of the detected infection region is represented by the scale bar on the right side. The radiologist or doctor will be able to determine the severity of the infected lung region using this method.

**Table 3:** Quantitative analysis of the proposed method.

	Lung segmentation (%)	Infection segmentation (%)
Accuracy (%)	98.07	99.20
Sensitivity (%)	93.47	83.33
Specificity (%)	98.64	99.84
Precision (%)	89.75	89.93
MCC (%)	90.52	81.02
Dice score (%)	92.46	82.00

**Table 4:** Comparison of the proposed method with current literature works.

Authors	Data set	Approaches used	Results
Zhou et al.^10^	Set1:100 CT images 60 patients with Covid-19	UNet + tverskyloss function	Dice Score—83.1%
	Set2: 9 volumes, total 829 slices		Sensitivity—86.7%
			Specificity—99.3%
Jin et al.^11^	1136 cases 723 positives	UNet+, CNN	Sensitivity—97.4%
			Specificity—92.2%
Amyar et al.^12^	1044 patients 449—COVID-19,	CNN, encoder and two decoder based architecture	Dice Score > 78.0%
	100—normal ones, 98—lung cancer,		
	397 with different kinds of pathology		
Wu et al.^13^	CT scan images from 750 cases	Encoder Decoder architecture	Dice Score—78.3%
	400—COVID-19.		
Yan et al.^15^	21,658 CT images	Deep CNN	Dice Score—72.6%
Fan et al.^14^	100 axial CT images COVID-19 for Inf-Net	Inf-Net,	Dice score—68.2%
	45 CT images for Semi-Inf-Net	Semi-Inf-Net	Sensitivity—69.2%
			Specificity—94.3%
			Dice score—73.9%
			Sensitivity—72.5%
			Specificiy—96.0%
Wang et al.^16^	558 CT images	Residual connection	Dice score—80.7%
Proposed	20 CT volumes	Cascaded 3D Unet, augmentation	Dice Score-82.0%
			Sensitivity—83.33%
			Specificity—99.84%

The performance analysis of the proposed method for lung segmentation and lung infection segmentation is shown in Table 3. Table 4 shows the comparative analysis of the proposed method with the methods in current literature.

Using UNet and the tversky loss function, Zhou et al.^10^ detect the contaminated zone. The studies are conducted on two data sets: set 1 consists of 100 axial CT images from 60 patients with Covid-19. Set 2 consists of 9 volumes with 829 slices, of which 373 slices have been evaluated and segmented as COVID-19 instances by a radiologist. They achieved a Dice score of 83.1%, a Sensitivity of 86.7%, and a Specificity of 99.3%, respectively. Jin et al.^11^ employed 1136 images, 723 of which were positives collected from five different hospitals, to develop their method. They used fully convolutional networks (FCN-8s), U-Net, V-Net, and 3D U-Net++ to analyse the results. The results show that the 3D UNet++ gives a dice coefficient of 0.754. Amyar et al.^12^ presented a three-arm architecture. Here the reconstruction and segmentation are done by an encoder and two decoders. A multi-layer perceptron network performs the classification, and they got a dice coefficient of 0.78.

The images are given to the classification system one by one in the method proposed by Wu et al.^13^. The patient is diagnosed with COVID-19 if the number of infected CT images exceeds a certain threshold. An activation map is also included in the classification model. An encoder-decoder method with enhanced feature modules is used for segmentation. On the segmentation test set, the segmentation method received a dice score of 78.3 %. The method by Fan et al.^14^, a parallel partial decoder is used to aggregate the high-level features and generate a global map. After that, implicit reverse attention and explicit edge attention are used to model the boundaries and enhance the representation. The Inf-Net with Res2Net gets a dice score, sensitivity, and specificity 68.2%, 69.2%, 94.3%, respectively. The Semi-Inf-Net got a dice, sensitivity, and specificity of 73.9%, 72.5%, 96.0%, respectively. The method proposed by Yan et al.^15^ uses extensive data set consist of 21,658 CT images annotated from 861 patients with confirmed COVID-19. They proposed a deep CNN with a feature variation block and Progressive Atrous Spatial Pyramid Pooling, got a Dice coefficient of 98.7% for lung segmentation and 72.6% for COVID-19 infection segmentation.

To deal with noisy images, Wang et al.^16^ proposed a noise-robust dice loss and a deep network called the COPLE-Net. Then these two are combined with an adaptive self-ensembling framework for training. As a result, they got a dice score of 80.72%. By comparing, the proposed method can segment the lung parenchyma and lung infections more accurately. The method got an Accuracy, Sensitivity, Specificity, Precision, MCC, and a Dice score of 98.07%, 93.47%, 98.64%, 89.75%, 90.52%, and 92.46% respectively for the lung parenchyma segmentation. The method got an Accuracy, Sensitivity, Specificity, Precision, MCC, and a Dice score of 99.20%, 83.33%, 99.84%, 89.93%, 81.02%, and 82.0% respectively for the lung infection segmentation. The proposed method can also distinguish the arteries and the lung infections correctly even though they have similar intensity values.

## Conclusion

To reduce the death rate and the spread of diseases, computer-aided diagnosis can be used to get the situation under control, as the entire world is in the grip of the COVID-19 pandemic. Here, we proposed a 3D cascaded UNet architecture to segment the infection lesion from the lung parenchyma. The majority of the studies employed a private data set; however, we conducted our study using a publicly available data set. The developed algorithm’s performance was evaluated by calculating Accuracy, Sensitivity, MCC, and Dice Score. We achieved good accuracy by combining fundamental image processing techniques with well-known CNN designs. Another advantage of the suggested method is that the learning happens step by step, i.e., first with the lung parenchyma volume segmentation then infected lung volume segmentation. This will increase the accuracy of the segmentation algorithm and reduce the complexity. As a result, there is no need to label the lung parenchyma volume when inputting the new patients’ CT volumes, which is a time-consuming task for the radiologist. This work allows the doctors to analyze the contaminated area in the whole CT volume based on the severity of the infection. Objects with low-intensity lung infections can also be detected using this method. This method outperforms the various intensity-based, region-based and active contour-based segmentation methods. As a future scope, we plan to distinguish the lung abnormalities such as lymphadenopathy and pleural effusion along with the COVID-19 infection. We also intend to acquire more 3D CT volumes so that the CNN can be better trained and implemented at a hospital to design all of the life-support systems ahead of time in such a troublesome situation.

## Data Availability

The data set used in this study is publically available one by Ma et al.
